# Three-Dimensional Morphometry Findings of the Proximal Humerus of the Indonesian Population

**DOI:** 10.7759/cureus.78181

**Published:** 2025-01-29

**Authors:** Iman W Aminata, Salman A Nizami

**Affiliations:** 1 Orthopaedics and Traumatology, Fatmawati General Hospital, Jakarta, IDN; 2 Faculty of Medicine, University of Indonesia, Jakarta, IDN

**Keywords:** ct scan, implant, indonesia, morphometry, proximal humerus, shoulder arthroplasty

## Abstract

Background

The design of implant components is the most critical prognostic factor in total shoulder arthroplasty as even minor discrepancies between the implant and the native anatomy can lead to failure. In this study, we aimed to describe the morphometry findings of the proximal humerus at our center, a national referral hospital located in the capital of Indonesia, serving a diverse population representing various Indonesian ethnicities.

Methodology

This observational, cross-sectional study included patients who had undergone upper extremity CT scans from January to December 2022. Demographic data such as age, sex, height, weight, body mass index, and ethnicity were collected from medical records. The measurements taken included the humeral head height (HHH), articular surface diameter (ASD), humeral head diameter (HHD), head-neck angle (inclination angle), and medial offset by CT scans.

Results

A total of 70 patients met the inclusion criteria. The average measurements were 14.3 ± 1.7 mm for HHH, 131.25° ± 5.15° for the head-neck angle (inclination angle), 40.50 ± 3.23 mm for the ASD, 44.86 ± 4.17 mm for the HHD, and 6.25 ± 1.93 mm for medial offset. HHH, ASD, and HHD showed significant differences between genders. Additionally, body height was significantly associated with HHH, ASD, and HHD. There was no significant measurement between ethnic groups in Indonesia.

Conclusions

A morphometric study of the proximal humerus revealed that the Indonesian population is vastly different than populations. Factors that need to be considered in selecting a shoulder implant include the patient’s height, gender, and race. There was a direct correlation between ASD, HHD, and HHH. Body height had a direct correlation with ASD, HHD, and HHH. The proximal humerus measurements in the Indonesian population were not significantly different between ethnic groups.

## Introduction

Shoulder arthroplasty has become a standard procedure for many shoulder pathologies, with prosthetic design being crucial in improving clinical outcomes. Even minor discrepancies between the prosthesis and native anatomy can significantly impact the success of total shoulder arthroplasty (TSA) [1]. Most prostheses were designed in Western countries based on the anatomical characteristics of Western populations, which may pose a risk for Indonesian individuals, ranked among the world’s top 10 shortest populations, of receiving prostheses that are not suitable for their stature. Due to limited advancements in prosthetic design in Indonesia, efforts have been made to create a prosthesis that matches the native geometry of the proximal humerus despite previous research indicating significant racial differences in anatomical measurements [2]. Anatomic features of the proximal humerus morphology have been investigated for specific populations [3-6]. However, studies on the morphometrics of the proximal humerus in Indonesia remain limited. This study aimed to describe the proximal humerus morphometrics at our center, a national referral hospital located in Indonesia’s capital city, which serves a diverse population representing various Indonesian ethnicities.

## Materials and methods

In this study, 70 upper extremity CT scans obtained between January and December 2022 from Fatmawati General Hospital’s Radiology Department were analyzed. The inclusion criteria for this study were skeletally mature patients between 18 and 80 years old with precise imaging of the proximal humerus. Patients with pre-existing conditions such as humeral fractures, humeral operative history, humeral infections, congenital disorders of bone growth, and humeral tumors were excluded because they can alter the native size of the proximal humerus area. In total, 70 patients who met the inclusion criteria were included in the study. Demographic data such as age, sex, body height, body weight, body mass index (BMI), and diagnosis were obtained from electronic medical records.

Image acquisition and three-dimensional model reconstruction of the proximal humerus

The CT scans were performed using a GE Revolution CT scanner with a 1 mm slice thickness. Serial axial scans were acquired and stored in the Digital Imaging and Communications in Medicine (DICOM) format. MIMICS software (Mimics Research 21.0, Materialise, Leuven, Belgium) was used to convert the scans into patient-specific three-dimensional (3D) computer models. Bone segmentation was performed using a single-threshold method, as previously described [7]. Two independent observers (SAN and IWA) took all measurements, and their readings were averaged for each measurement.

**Figure 1:**
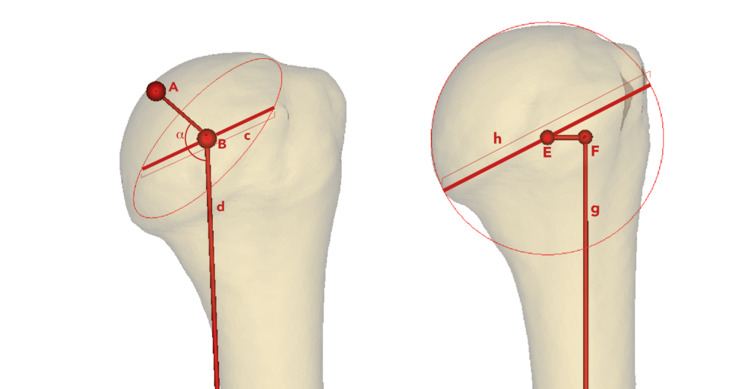
Illustration of the morphometric measurements. Point A is the peak of the humeral head; point B is the center of the articular surface/neck plane; line c is the articular surface, diameter; line d is a guide line, parallel to the humeral axis, attached to point B; angle α is the neck-shaft angle (also called inclination angle); line AB is the humeral head height (also called humeral head thickness); point E is the center of the humeral head sphere; line g is the humeral shaft axis; point F is the edge of line g; line h is the humeral head diameter; line EF is the medial offset. The left image is the anterior view of the left humerus. The right image is the posterior view of the left humerus. Image credit: Salman A Nizami

The morphometric parameters measured in this study included the neck-shaft angle (inclination angle), articular surface diameter (ASD), humeral head height (HHH), humeral head diameter (HHD), and medial offset. An illustration of these measurements is provided in Figure 1.

**Figure 2:**
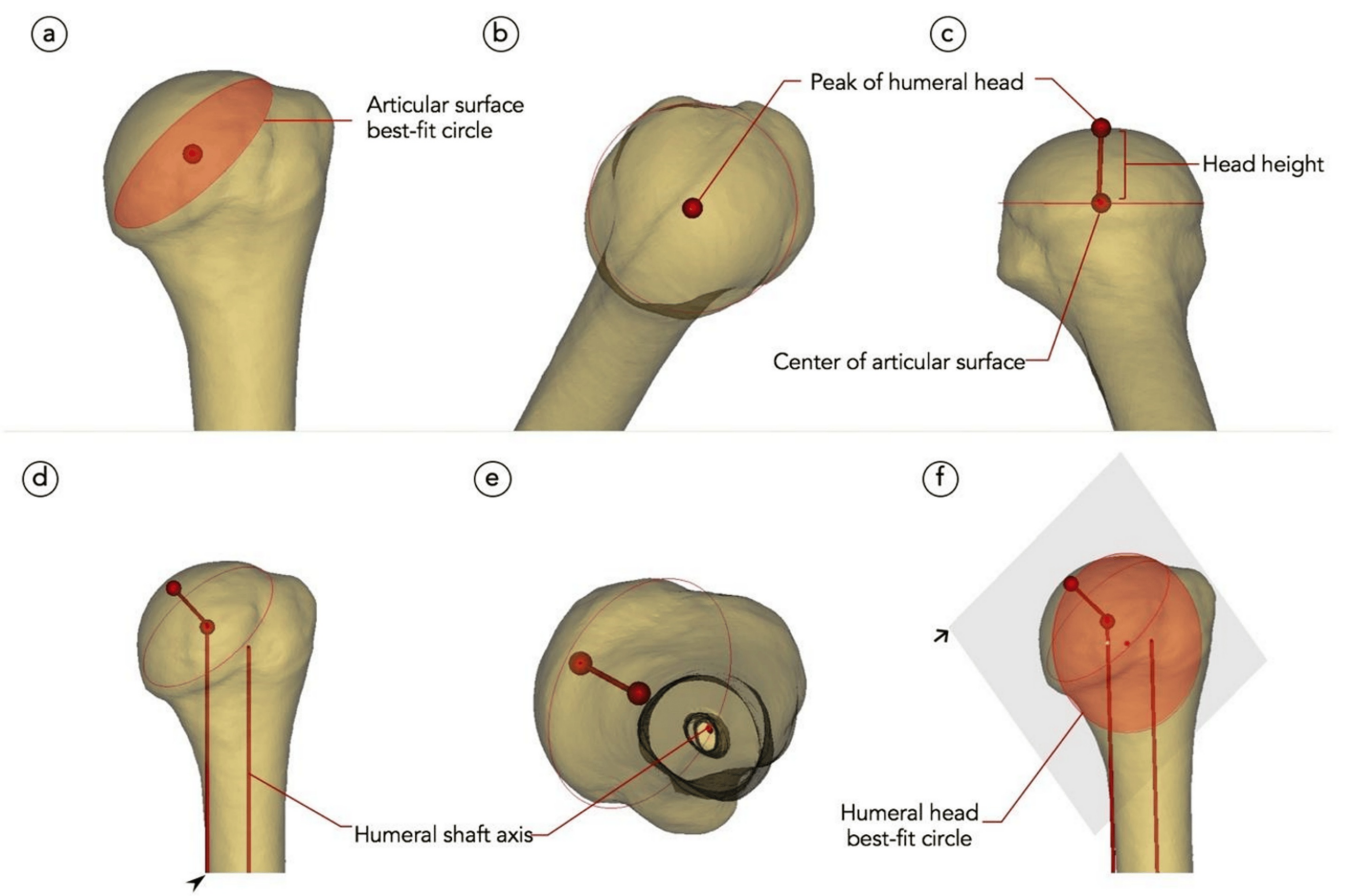
Stepwise illustration of the measurement process (a-f). Black arrowhead: a guide line parallel to the humeral shaft axis (line d in Figure 1). Black arrow: plane containing the peak of the humeral head, center of the articular surface, and humeral head best-fit circle. Image credit: Salman A Nizami

The articular surface was first determined by defining the base of the humeral head sphere in reference to the anatomic neck plane. Three points were selected along the ridge of the anatomical neck, such as a point on the junction between the articular surface and greater tuberosity and two points on the margin of the anteroinferior and posteroinferior part of the articular surface, similar to the methods reported by Matsuki et al. [8]. The ASD was determined by creating a circle that best fit the region representing the articular surface (Figure 2a). HHH was defined as the distance between the center of the articular surface circle and the top of the humeral head (Figures 2b, 2c). The humeral shaft axis was defined as a line passing through the center of the metaphyseal cylinder (Figures 2d, 2e). The head-neck angle was defined as the angle between the HHH and the humeral shaft axis. HHD was defined as the circle’s diameter best fitting the humeral head sphere (Figure 2f). Medial offset was defined as the distance between the humeral shaft axis and the center of the HHD.

Statistical analysis

Statistical analysis was conducted using SPSS Statistics version 25 software (IBM Corp., Armonk, NY, USA), while descriptive statistics were compiled in Microsoft Excel (Microsoft Corp., Redmond, WA, USA). The Kolmogorov-Smirnov test was employed to assess the distribution of all parameters. Comparisons based on sex and humeral side were analyzed using two-tailed Student’s t-tests for normally distributed data or the Mann-Whitney test for skewed data. Correlations among patient’s characteristics (body weight, body height, and BMI) and proximal humerus morphometry (head-neck angle, HHH, ASD, HHD, and medial offset) were analyzed using Pearson’s correlation tests for normally distributed data or Spearman’s rank test for skewed data. The relationship between ethnicity and all morphometric parameters was assessed using a one-way analysis of variance or the Kruskal-Wallis test. A significance level of 0.05 was set for all analyses.

## Results

Table 1 shows the characteristics of the patients included in the study. We analyzed a total of 70 upper extremity CT scans. The majority of participants were from Jakarta, where the investigation was conducted. In the study, more than 10 ethnic groups participated to assess whether there were differences between ethnicities. Lung tumors were the most common underlying disease in this population. Patients ranged from 23 to 76 years old. The most common underlying disease was a tumor, two of which were of bone origin. None of the two bone tumors were located at the humerus.

**Table 1:** Demographic characteristics of the study participants (n = 70).

Characteristic	Number (%)/Average
Age	51.11 ± 12.98
Sex	
Male	24 (34.28%)
Female	46 (65.71%)
Body height (cm)	157.35 ± 12.98
Body weight (kg)	52.44 ± 9.83
Body mass index (kg/m^2^)	21.23 ± 4.09
Humerus analyzed	
Right	35 (50%)
Left	35 (50%)
Dexterity	
Right	35 (50%)
Left	35 (50%)
Ethnicities	
Pangkal Pinang	2.85%
North Sumatera	4.28%
Riau	1.42%
West Sumatera	2.85%
South Sumatera	1.42%
West Java	27.14%
Jakarta	41.42%
Central Java	11.42%
East Java	2.85%
South Sulawesi	1.42%
Papua	2.85%

Table 2 shows the overall result of the morphometric measurement. The results of proximal humerus measurements from the population in Indonesia were relatively small.

**Table 2:** Morphometric measurement findings.

Morphometric measurements	Minimum	Maximum	Mean	SD
Neck-neck angle (degrees)	114.20	143.07	131.25	5.15
Humeral head height (mm)	10.4	17.5	14.31	1.7
Articular surface diameter (mm)	35.3	47.2	40.50	3.23
Humeral head diameter (mm)	38.2	58.1	44.86	4.17
Medial offset (mm)	1.1	11.2	6.25	1.93

Table 3 shows the measurements in groups based on sex. According to these results, both men and women have significant differences in the size of the proximal humerus; hence, gender should be considered while selecting implants.

**Table 3:** Comparison of morphometric measurements between sexes (t-test).

Morphometric measurements	Mean	SD	t-value	P-value
Head-neck angle (inclination angle)				
Male	132.63	6.67	0.28	0.77
Female	131.13	4.82		
Humeral head height				
Male	15.30	1.37	3.87	<0.001
Female	13.80	1.61		
Articular surface diameter				
Male	43.83	2.26	9.26	<0.001
Female	38.16	2.7		
Humeral head diameter				
Male	49.08	3.12	8.92	<0.001
Female	42.21	3.7		
Medial offset				
Male	6.27	2.28	0.05	0.95
Female	6.24	1.75		

Table 4 shows the relationship between patient profile and proximal humerus morphometric measurements. From Table 4, it can be concluded that height was the only factor in the patient’s body profile to be considered in selecting implants.

**Table 4:** Correlation analysis (Pearson’s test) between body profile and proximal humerus morphometry.

Morphometry	Height (P-value)	Body weight (P-value)	Body mass index (P-value)
Head-neck angle (inclination angle)	0.13 (0.08)	0.09 (0.27)	0.01 (0.31)
Humeral head height	0.3 (<0.001)	0.12 (0.11)	0.01 (0.21)
Articular surface diameter	0.64 (0.03)	0.33 (0.08)	0.03 (0.29)
Humeral head diameter	0.62 (0.02)	0.33 (0.12)	0.03 (0.31)
Medial offset	0.07 (0.09)	0.18 (0.18)	0.11 (0.22)

The morphometric analysis of the proximal humerus across different ethnic groups revealed no statistically significant differences in key measurements (Table 5). These findings indicate that the proximal humeral morphometry for the measured parameters was likely consistent across ethnicities.

**Table 5:** Comparison analysis of proximal humerus using a one-way analysis of variance of morphometric measurements among Indonesian ethnic groups.

Morphometry among ethnicities	F-value	P-value
Head-neck angle (inclination angle)	0.92	0.52
Humeral head height	1.38	0.20
Articular surface diameter	1.01	0.44
Humeral head diameter	0.80	0.62
Medial offset	1.13	0.35

Correlation analysis uncovered several directly related variables. An increase in HHD was proportionally related to an increase in HHH and ASD (r = 0.528, p < 0.001, and r = 0.916, p < 0.001, respectively). Moreover, ASD was also found to have a strong linear correlation with HHH (r = 0.679, p < 0.001).

## Discussion

The humeral head anatomic restoration is essential for a successful shoulder replacement. Joint kinematics and the results of an anatomic shoulder arthroplasty can be impacted by an implant that is not in harmony with the natural anatomy. Over the past 20 years, the design and fixation of the humeral component have changed to either adapt implants to soft tissue conditions or enable anatomic restoration of premorbid proximal humerus anatomy [9].

A reduction in range of motion after surgery may arise from overstuffing the joint and over-tensioning soft tissues when the humeral head implant is thicker than the native bone size. However, as the size of the humeral head implant decreases, the glenoid may experience increased tuberosity impingement and point loading. It is advised that to produce appropriate tension on the posterior capsule and cuff, a thicker humeral head size may be necessary for arthritic shoulders with extensive posterior subluxation. However, to prevent stiffness in avascular necrosis, downsizing could be required [9-11]. The doctor’s experience and the size of the native bone are very important in determining the size of the implant.

In a study conducted in Australia, the implant product with a size of HHD of more than 50 mm had good outcomes for the Australian population, while the failure rate was very high for a diameter of less than 44 mm [12]. From the data we obtained, the size of Indonesian HHD was 38-58 mm, implying that different populations need different specifications of the implant.

Both surgeons and implant manufacturers should find our study conclusions valuable when making selections. The suitable implant size, together with the corresponding thickness combinations, should be available. Two implant systems (Implantcast, Germany, and Corentec, South Korea) are accessible in Indonesia. Only Corentec implant systems have humeral head sizes <40 mm that are appropriate for the Indonesian population with a humeral head size of 43.8 (38-58 mm). However, even the thickness combinations (15-21 mm) were larger for the Indonesian population (10-18 mm). This mismatch between the implant and native bone anatomy is crucial because the implant could shift the center of rotation and may alter the lever arm of the rotator cuff. This could lead to secondary rotator cuff insufficiency, which requires revision in the form of reverse shoulder arthroplasty. Therefore, some adjustment to the size of the implant is needed to prevent TSA failure [13].

The study findings are similar to a study conducted in the Thai population reported by Aroonjarattham et al. [14]. Despite the similar measurement methods used, the study was performed in cadaver specimens. The Thai population’s mean head-neck angle was 127.6°, mean HHH was 14.8 mm, mean ASD was 40.5 mm, and mean HHD was 42.6 mm [14]. Another study by Zhang et al. in the Chinese population showed larger measurement results, with mean HHH and ASD of 16.9 mm and 42.9 mm, respectively [6]. The neck-shaft angle (133°) of the Chinese population was comparable to our study [6]. The proximal humerus size of the Indonesian population was smaller than the Chinese population but larger than the Thai population. In comparison to other Asian, European, and American populations, the measurements of the Indonesian population were smaller for all parameters [5,15-19].

The most significant finding was the correlation between ASD and HHD, followed by the correlation between HHH and both ASD and HHD. Inyang et al. reported similar correlations in their study that measured South African and Swiss populations, with the strongest correlation observed between HHD and ASD [20].

Several methods are available to measure the morphometrics of the proximal humerus. The most conventional approach involves direct measurements of cadaveric humeri specimens. However, advances in imaging technology have enabled in vivo measurements through direct two-dimensional (2D) imaging techniques (such as plain radiographs, CT scans, and MRIs), which can then be used for 3D modeling [17,19,21]. Prior studies indicate that measurements made on 3D CT scans are reproducible with better precision than those obtained from plain radiographs [21]. Overall, 3D models generated from CT scans are comparable regardless of the segmentation method used [7]. Measurements performed by Matsuki et al. and DeLude et al. presented axis formation as achievable regardless of the original CT scan orientation [8,16]. The steps performed in acquiring the morphometric variables may differ between studies. However, some variables have a common method of measurement, such as the definition of the neck plane, humeral axis, HHH, and neck-shaft angle [5,15-21].

There are notable differences in bone geometry among ethnicities. For instance, hip axis length is shorter in Black and South Asian men compared to White men, while South Asians exhibit smaller bones at the metaphysis and diaphysis of the radius. In contrast, our study found no significant size differences in proximal humerus among the various ethnic groups in Indonesia [22].

This study was performed using CT scans of living patients. To our knowledge, this is the first study to describe the morphometric profile of the proximal humerus of the Indonesian population. The limitation of this study is the inclusion of a limited number of ethnic groups in Indonesia, which has more than 600 ethnic groups. Further studies are needed regarding the biomechanics of special implant sizes for the population in Indonesia.

## Conclusions

A morphometric study of the proximal humerus revealed that the morphometric measurements of the Indonesian population tend to be on the lower side when compared to the Western counterparts owing to ethnically smaller bone structure. Factors that need to be considered in selecting shoulder implants include the patient’s height, gender, and race. There was a direct correlation between ASD, HHD, and HHH. Body height had a direct correlation with ASD, HHD, and HHH. The proximal humerus measurements in the Indonesian population were not significantly different between ethnic groups.
